# Comparative efficacy and safety of dolutegravir relative to common core agents in treatment-naïve patients infected with HIV-1: a systematic review and network meta-analysis

**DOI:** 10.1186/s12879-019-3975-6

**Published:** 2019-05-30

**Authors:** Sonya J. Snedecor, Matthew Radford, David Kratochvil, Richard Grove, Yogesh S. Punekar

**Affiliations:** 1 Pharmerit International, Bethesda, MD USA; 2 ViiV Healthcare, GSK House, 980 Great West Rd, Brentford, Middlesex TW8 9GS UK; 3 GSK, Brentford, Middlesex UK

**Keywords:** Antiretroviral therapy, Dolutegravir, HIV-1, Network meta-analysis, Systematic review, Treatment-naïve, Integrase strand inhibitors, Non-nucleoside reverse transcriptase inhibitor, Protease inhibitor

## Abstract

**Background:**

Network meta-analyses (NMAs) provide comparative treatment effects estimates in the absence of head-to-head randomized controlled trials (RCTs). This NMA compared the efficacy and safety of dolutegravir (DTG) with other recommended or commonly used core antiretroviral agents.

**Methods:**

A systematic review identified phase 3/4 RCTs in treatment-naïve patients with HIV-1 receiving core agents: ritonavir-boosted protease inhibitors (PIs), non-nucleoside reverse transcriptase inhibitors (NNRTIs), or integrase strand inhibitors (INSTIs). Efficacy (virologic suppression [VS], CD4^+^ cell count change from baseline) and safety (adverse events [AEs], discontinuations, discontinuation due to AEs, lipid changes) were analyzed at Week 48 using Bayesian NMA methodology, which allowed calculation of probabilistic results. Subgroup analyses were conducted for VS (baseline viral load [VL] ≤/> 100,000copies/mL, ≤/> 500,000copies/mL; baseline CD4^+^ ≤/>200cells/μL). Results were adjusted for the nucleoside/nucleotide reverse transcriptase inhibitors (NRTI) combined with the core agent (except subgroup analyses).

**Results:**

The NMA included 36 studies; 2 additional studies were included in subgroup analyses only. Odds of achieving VS with DTG were statistically superior to PIs (odds ratios [ORs] 1.78–2.59) and NNRTIs (ORs 1.51–1.86), and similar but numerically higher than other INSTIs. CD4^+^ count increase was significantly greater with DTG than PIs (difference: 23.63–31.47 cells/μL) and efavirenz (difference: 34.54 cells/μL), and similar to other core agents. INSTIs were more likely to result in patients achieving VS versus PIs (probability: 76–100%) and NNRTIs (probability: 50–100%), and a greater CD4^+^ count increase versus PIs (probability: 72–100%) and NNRTIs (probability: 60–100%). DTG was more likely to result in patients achieving VS (probability: 94–100%), and a greater CD4^+^ count increase (probability: 53–100%) versus other core agents, including INSTIs (probability: 94–97% and 53–93%, respectively). Safety outcomes with DTG were generally similar to other core agents. In patients with baseline VL > 100,000copies/mL or ≤ 200 CD4^+^cells/μL (18 studies), odds of achieving VS with DTG were superior or similar to other core agents.

**Conclusion:**

INSTI core agents had superior efficacy and similar safety to PIs and NNRTIs at Week 48 in treatment-naïve patients with HIV-1, with DTG being among the most efficacious, including in patients with baseline VL > 100,000copies/mL or ≤ 200 CD4^+^cells/μL, who can be difficult to treat.

**Electronic supplementary material:**

The online version of this article (10.1186/s12879-019-3975-6) contains supplementary material, which is available to authorized users.

## Background

The emergence of combination antiretroviral therapy (cART) dramatically improved outcomes for patients with human immunodeficiency virus (HIV) infection, transforming it into a manageable chronic condition with a life expectancy similar to that in the general population [1, 2]. Generally, cART results in durable virologic suppression (VS) and CD4^+^ cell repletion, with reduced morbidity, decreased hospitalization rates, and reduced mortality, in addition to preventing HIV transmission [1, 3–5]. However, all ARTs are associated with adverse effects, which are the most common reasons for switching or discontinuing therapy and for treatment non-adherence [6].

Current guidelines from the World Health Organization (WHO), the US Department of Health and Human Services (DHHS), and the European AIDS Clinical Society (EACS) recommend first-line cART comprising a core agent (integrase strand inhibitor [INSTI], boosted protease inhibitor [PI], or non-nucleoside reverse transcriptase inhibitor [NNRTI]) in combination with two nucleoside/nucleotide reverse transcriptase inhibitors (NRTIs) for treatment-naïve patients with HIV-1 [7–10]. Recommended or commonly used core agents include the INSTIs bictegravir (BIC), dolutegravir (DTG), cobicistat-boosted elvitegravir (EVG/c), and raltegravir (RAL); the ritonavir-boosted PIs atazanavir (ATV/r), darunavir (DRV/r), and lopinavir (LPV/r); or the NNRTIs efavirenz (EFV) and rilpivirine (RPV).

Network meta-analyses (NMAs) allow the evaluation of the comparative efficacy and safety of the increasing numbers of treatment choices for treatment-naïve patients with HIV-1 to be evaluated in the absence of head-to-head clinical studies. An NMA conducted in 2016 compared INSTIs with EFV (the preferred core agent at that time according to the WHO [9]) in treatment-naïve patients with HIV-1 and found a clear hierarchy within the INSTI class, with DTG being the most efficacious followed by RAL, then EVG/c. DTG was statistically superior to EFV, ATV/r, DRV/r, LPV/r, NVP, and RPV for VS at Week 48, and to EFV, EVG/c, ATV/r, DRV/r, LPV/r, and NVP for VS at Week 96. DTG was also statistically superior to EFV, EVG/c, ATV/r, LPV/r, and NVP with regards to rates of discontinuation due to adverse events (AEs) [11]. These results were consistent with those of an earlier NMA conducted in 2013, in which DTG had similar or superior efficacy to the recommended core agents at that time (EVG/c, RAL, ATV/r, DRV/r, LPV/r, EFV, and RPV) [12]*.*

DTG is an INSTI approved for the treatment of HIV-1 in combination with other antiretroviral agents [13, 14]. DTG is recommended once daily for adult patients infected with HIV-1 who do not have documented or clinically suspected resistance to INSTIs [13, 14]. In randomized controlled trials (RCTs) DTG had a higher barrier to resistance than RAL [15], was superior to once-daily EFV and once-daily DRV/r, and non-inferior to twice-daily RAL for the treatment of treatment-naïve patients with HIV-1 [15–18]. As a result of the 2016 NMA that compared INSTIs with EFV [11], the WHO now recommends a DTG-based regimen as a preferred first-line therapy for treatment-naïve patients with HIV-1 [10]. The EACS and the US DHHS now recommend INSTIs, including DTG, as first-line core agents for treatment-naïve patients with HIV-1 [7, 8].

With the publication of new data and updates to guideline recommendations, we conducted a systematic review and NMA to evaluate DTG against other guideline-recommended core agents in treatment-naïve patients with HIV-1 infection, to update the earlier (2013) NMA [12].

## Methods

### Study identification

A systematic search of PubMed/MEDLINE, Embase, and Cochrane databases was undertaken in December 2016 and September 2017 to update the original search conducted in 2013 [12] to identify RCTs evaluating the efficacy and/or safety of cART in treatment-naïve patients with HIV-1. The search strategy for PubMed and Embase is shown in Additional file 1: Table S1. Resources used to identify these RCTs also included the National Institute of Health clinical trial (NCT) registry database (www.clinicaltrials.gov), US Food and Drug Administration (FDA) approval summaries, and European Medicines Agency (EMA) and European Public Assessment Reports (EPAR) scientific discussions and package inserts. Additional records were identified through manual searching. Study selection was performed using three sequential steps: 1) abstracts identified from the electronic and manual searches were archived into a master bibliography management database; 2) initial review and selection of study titles/abstracts by two independent reviewers; 3) full-text review and selection of final study sample for data extraction by two different independent reviewers. Any discrepancies between the reviewers were resolved by consensus. Study data were extracted into a structured Microsoft Access database by at least two independent reviewers and reconciled for accuracy.

Treatments of interest were the following guideline-recommended core agents: INSTIs (DTG, BIC, EVG/c, RAL), ritonavir-boosted PIs (ATV/r, DRV/r), and NNRTIs (EFV, RPV) [7, 8, 10], plus LPV/r, i.e. all core agents recommended at the time of the original analysis [12], and additional recommendations in current guidelines [7, 8]. Although LPV/r is no longer recommended as a first-line treatment option, it was retained for consistency with prior NMAs [11, 12]. Further information regarding these core agents can be found in the EMA EPARs (www.ema.europa.eu/en) or the FDA prescribing information (www.accessdata.fda.gov/scripts/cder/daf/index.cfm). Publications were included if they were phase 3/4 RCTs in, or including a subgroup of, treatment-naïve adults or adolescents (≥13 years of age) with HIV-1 infection; published in the English language; including one of the core agents of interest in combination with two NRTIs and at least one comparator; and reporting at least one of the efficacy or safety outcomes of interest. Studies in which any two of these core agents were compared were included in the analyses. Studies in which one arm was a treatment of interest and another was a “connector” – not a treatment of interest, but one that had been studied in head-to-head comparisons with two or more core agents of interest – were also included, in accordance with published guidelines for NMAs [19, 20]. Data from these connector studies help to strengthen the indirect estimates of the NMA. Studies investigating various dosages of a core agent, with a sample size < 50, or conducted in pediatric populations (< 13 years of age) were excluded.

The Preferred Reporting Items for Systematic Reviews and Meta-Analyses (PRISMA) guidelines were followed in all phases of the study [21].

### Outcomes

#### Efficacy

Efficacy outcomes were the proportion of patients with VS at Week 48 and the change from baseline in CD4^+^ cell count at Week 48. In accordance with FDA guidance [22], the following outcomes were considered representative of VS (in order of preference): FDA Snapshot-50, time to loss of virologic response-50 (TLOVR-50), confirmed virologic response-50 (CVR-50), and HIV RNA < 50 copies/mL.

#### Safety

Safety outcomes were the proportion of patients with any grade AE (individual AEs were not analyzed separately), overall discontinuations, discontinuation due to AEs, and lipid changes – increases from baseline in total cholesterol [TC], high-density lipoprotein [HDL], low-density lipoprotein [LDL], and triglycerides [TG].

#### Subgroups

Analysis of efficacy and safety outcomes in subgroups of patients with baseline viral load (VL) ≤100,000 and > 100,000, and ≤ 500,000 and > 500,000 RNA copies/mL, and with baseline CD4^+^ cell count ≤200 and > 200 cells/μL, were also planned (secondary objective).

### Data analysis

The NMA was conducted using a Bayesian analysis framework to generate estimates of relative treatment outcomes [23, 24] using WinBUGS (version 1.4.3). The efficacy and safety of core treatments of interest are reported relative to DTG. For each outcome, a fixed-effect (FE) and random-effect (RE) model was evaluated. The Deviance Information Criterion (DIC) was used to determine the better fit between the FE and RE models. Heterogeneity in the treatment effects was assessed and a network inconsistency model [20] was used to evaluate the network inconsistency of the efficacy outcomes (see Additional file 1).

Models were constructed using guidance from the United Kingdom National Institute for Health and Care Excellence (NICE) technical support document [19], modified to include a parameter to adjust for the NRTI used in combination with the core agent. Both NRTI-adjusted and unadjusted analyses were conducted. In the NRTI-adjusted analyses, NRTIs were grouped into three categories: abacavir/lamivudine (ABC/3TC), tenofovir disoproxil (or alafenamide) fumarate/emtricitabine (TD[A]F/FTC), or any other NRTI combination (Other). Analyses were carried out including and excluding connector studies. Vague prior distributions (e.g. normal with mean 0 and variance 10^5^) on model parameters were used so that outcomes would be determined only by data from the RCTs. Posterior outcome distributions were based on at least 20,000 simulations after a burn-in of at least 10,000.

Treatment effects for binary outcomes such as VS, AEs, and discontinuations were modeled using binomial likelihood and logit link function to estimate the odds ratios (OR) for VS between the treatments. Treatment effects for continuous outcomes (changes in CD4^+^ cell count and lipid levels) were modeled using a normal likelihood and identity link function to estimate the difference in the mean changes from baseline to Week 48 between the treatments of interest. Results were expressed as the median (50th percentile) of the posterior distribution of the treatment effect and 95% credible interval (CrI) – the 2.5th and 97.5th percentiles of the posterior distribution samples (i.e. representing the 95% probability that the parameter falls within this range). The Bayesian NMA methodology also allowed for estimates of the probability that one treatment is better than another to be calculated. As, by their nature, inferences from Bayesian analyses do not require adjustment for multiple comparisons [25], no adjustments for multiplicity were made.

### Data quality assessments

The quality of the studies selected was assessed based on study design, confounders, blinding, data collection methods, withdrawals, and dropouts, using the Effective Public Health Practice Project Quality Assessment (EPHPP) tool [26]. The quality of each comparison was scored according to the Grading of Recommendations Assessment, Development and Evaluation (GRADE) approach [27, 28] (see Additional file 1 for an overview of the GRADE algorithm). The GRADE approach provides a rating for the quality of the estimates of effect for a specific treatment comparison based on supporting direct and indirect evidence (possible ratings are High, Moderate, Low, or Very Low).

## Results

### Studies included

**Fig. 1:**
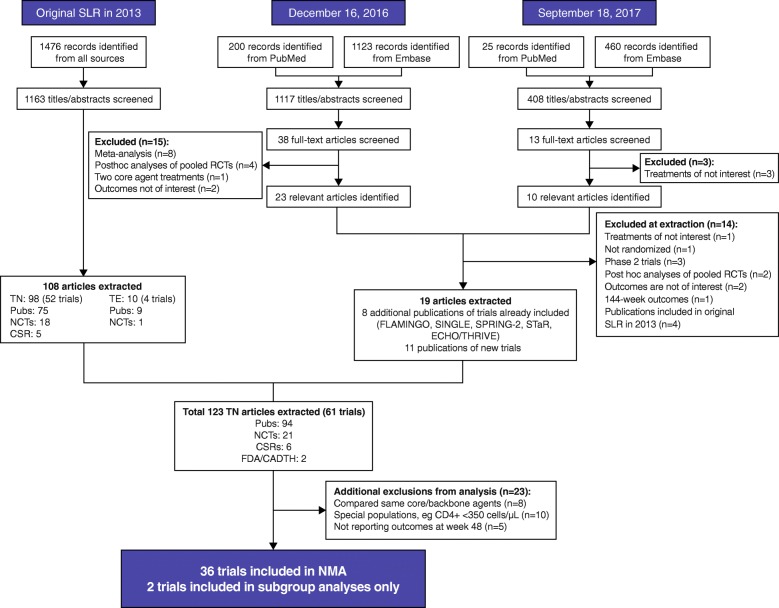
PRISMA flowchart of systemic literature review update. CADTH, Canadian Agency for Drugs and Technologies in Health; CSR, clinical study report; FDA, Food and Drug Administration; NCT, National Institute of Health clinical trial results published on ClinicalTrials.gov; NMA, network meta-analysis; PRISMA, Preferred Reporting Items for Systematic Reviews and Meta-Analyses; Pubs, published articles; RCT, randomized controlled trial; SLR, systemic literature review; TE, treatment experienced; TN, treatment naïve

In total, 2688 records were identified and screened; data from 123 records were documented that included 61 unique studies (Fig. 1). After data extraction, 23 studies were excluded, including those comparing the same core agent/NRTI combinations (*n* = 8) [29–37], special HIV populations (patients coinfected with tuberculosis or with specific CD4^+^ cell requirements other than < 200 cells/μL; n = 8) [38–49], conducted in subgroup of interest (CD4^+^ < 200 cells/μL) but not reporting outcomes of interest (*n* = 2) [50, 51], and outcomes not reported at Week 48 (*n* = 5) [52–56]. Overall, 36 studies involving 19,874 patients were included in the NMA (see Additional file 1: Table S2) [16, 18, 57–90]. The authors became aware of the availability of additional safety data for a previously identified study (GS-US-380-490) after the end of the systematic literature search window [91]. These data were included in the NMA to inform the safety analyses. An additional two studies comprising 309 patients were included in the subgroup analysis only [92, 93].

All studies included in the NMA were similar with respect to patient characteristics and inclusion/exclusion criteria [16, 18, 57–90, 92, 93]. The NMA inputs can be found in Additional file 1: Table S2. All but one study included in the NMA had an EPHPP rating of strong (*n* = 14) or moderate (*n* = 21) (see Additional file 1: Table S3). The open-label (not blinded) nature of the studies was the main factor contributing to the high number of moderately rated studies.

**Fig. 2:**
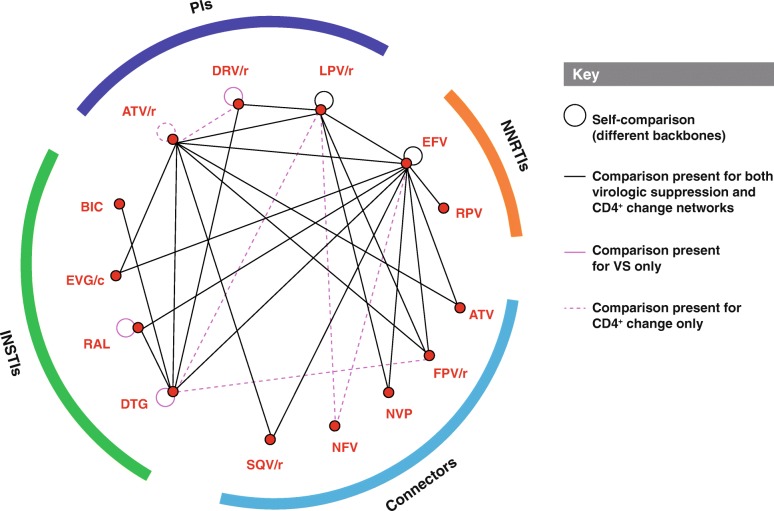
NMA Network of treatment comparisons for efficacy outcomes contained within the identified clinical studies. The major classes of agents analyzed in the selected trials for NMA are indicated along the perimeter of the figure: NNRTIs in orange, INSTIs in green, PIs in purple and connectors in blue. Black lines connecting each of the treatments of interest (red dots) represent a publication or clinical trial containing those 2 agents of interest. A connector is a treatment not of interest that is compared with at least two different treatments of interest that are included in the analysis to provide additional data. ATV, atazanavir; ATV/r, ritonavir-boosted atazanavir; BIC, bictegravir; DTG, dolutegravir; DRV/r, ritonavir-boosted darunavir; EFV, efavirenz; EVG/c, cobicistat-boosted elvitegravir; FPV/r, ritonavir-boosted fosamprenavir; INSTIs, Integrase strand inhibitors; LPV/r, lopinavir-boosted ritonavir; NFV, nelfinavir; NMA, network meta-analysis; NNRTIs, non-nucleoside reverse transcriptase inhibitors; PIs, protease inhibitors; RAL, raltegravir; RPV, rilpivirine; SQV/r, ritonavir-boosted saquinavir; VS, virologic suppression

The network of treatment comparisons for the efficacy outcomes is shown in Fig. 2. The following connector core agents were included within the network: atazanavir (ATV), ritonavir-boosted fosamprenavir (FPV/r), nelfinavir (NFV), nevirapine (NVP), and ritonavir-boosted saquinavir (SQV/r).

### Efficacy

**Fig. 3:**
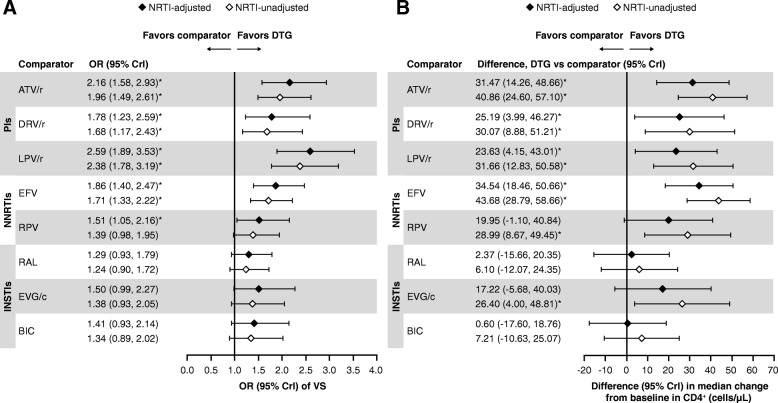
**a** VS and **b** median CD4^+^ CFB at Week 48 with DTG versus comparators. FE model. *Indicates treatment comparisons are significantly different. ATV/r, ritonavir-boosted atazanavir; BIC, bictegravir; CFB, change from baseline; Crl, credible interval; DTG, dolutegravir; DRV/r, ritonavir-boosted darunavir; EFV, efavirenz; EVG/c, cobicistat-boosted elvitegravir; FE, fixed effect; INSTIs, Integrase strand inhibitors; LPV/r, lopinavir-boosted ritonavir; NNRTIs, non-nucleoside reverse transcriptase inhibitors; NRTI, nucleoside/nucleotide reverse transcriptase inhibitor; OR, odds ratio; PIs, protease inhibitors; RAL, raltegravir; RPV, rilpivirine; VS, virologic suppression

Based on model diagnostics [94], the FE model was used for the primary interpretation of efficacy outcomes. Results of NRTI-adjusted efficacy analyses with connectors using the FE model are presented below. The results of analyses using NRTI-unadjusted FE models (Fig. 3a and b) and both NRTI-adjusted and unadjusted RE models (data not shown) were generally consistent with those using the NRTI-adjusted FE models. Similarly, analyses without connector studies (data not shown) yielded generally consistent results to the analyses with connectors presented here. The quality of the NMA comparisons, based on the GRADE assessment, are shown in Additional file 1: Table S4. No meaningful heterogeneity or substantial inconsistency were observed between the NMA and the direct evidence; any heterogeneity observed was due to small sample sizes (see Additional file 1).

#### VS at Week 48

**Fig. 4:**
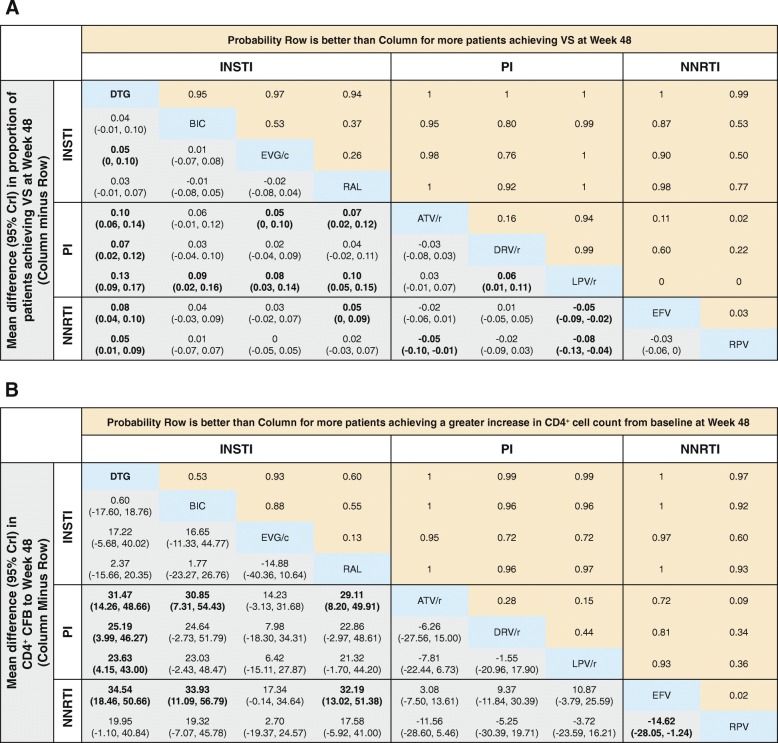
(**a**) VS and (**b**) CD4^+^ CFB at Week 48 with DTG versus comparators

The odds of achieving VS at Week 48 were statistically superior for DTG versus all ritonavir-boosted PIs (ORs 1.78–2.59) and NNRTIs (ORs 1.51–1.86), and numerically higher but not significantly different from other INSTI core agents (Fig. 3a). The probability that treatment with an INSTI core agent would result in patients achieving VS at Week 48 ranged from 76 to 100% versus ritonavir-boosted PIs, and 50 to 100% versus NNRTIs (Fig. 4a). Amongst INSTIs, patients treated with DTG were more likely to achieve VS at Week 48 was 94–97% (Fig. 4a).

#### Change in CD4^+^ cell count from baseline at Week 48

Treatment with DTG resulted in a significantly greater increase in mean CD4^+^ cell count from baseline at Week 48 than all ritonavir-boosted PIs (23.63–31.47 cells/μL) and EFV (34.54 cells/μL), and was similar to RPV and other INSTIs core agents (Fig. 3b and Fig. 4b). The probability that patients treated with INSTI core agents would achieve higher mean CD4^+^ cell counts at Week 48 ranged from 72 to 100% versus ritonavir-boosted PIs, and from 60 to 100% versus NNRTIs (Fig. 4b). Patients treated with DTG had higher probability of achieving a greater increase in mean CD4^+^ cell count at Week 48 compared with other INSTIs (53–93%) with differences in CD4^+^ cell counts between DTG and other core agents ranging from 0.60 cells/μL to 34.54 cells/μL (Fig. 4b).

### Safety

**Fig. 5:**
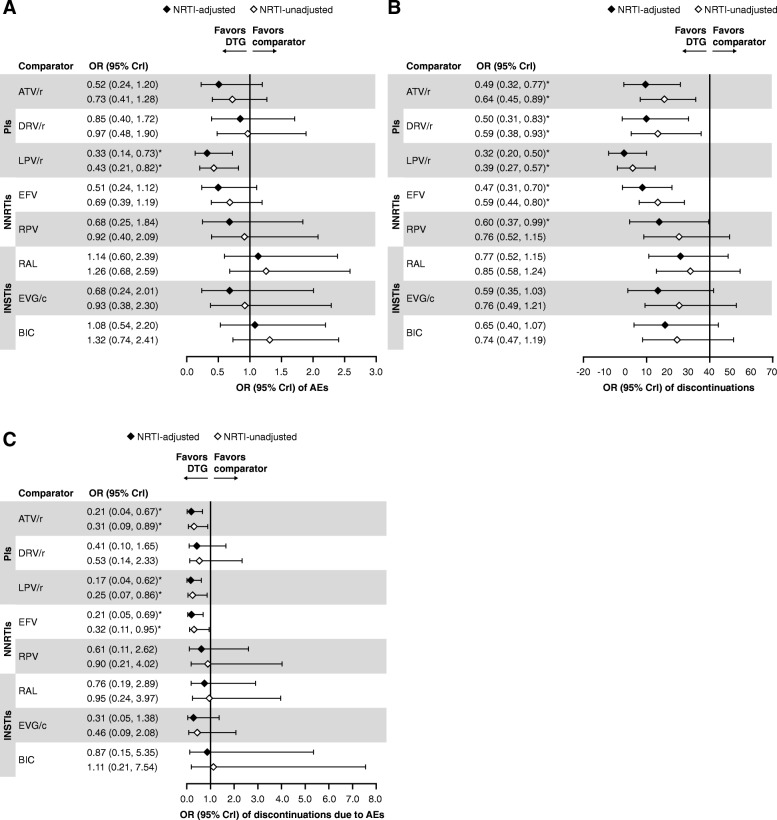
**a** AEs, **b** discontinuations, and **c** discontinuations due to AEs with DTG versus comparators. RE model. *Indicates treatment comparisons are significantly different. AEs, adverse effects; ATV/r, ritonavir-boosted atazanavir; BIC, bictegravir; Crl, credible interval; DTG, dolutegravir; DRV/r, ritonavir-boosted darunavir; EFV, efavirenz; EVG/c, cobicistat-boosted elvitegravir; INSTIs, Integrase strand inhibitors; LPV/r, lopinavir-boosted ritonavir; NNRTIs, non-nucleoside reverse transcriptase inhibitors; NRTI, nucleoside/nucleotide reverse transcriptase inhibitor; OR, odds ratio; PIs, protease inhibitors; RAL, raltegravir; RE, random effect; RPV, rilpivirine

**Fig. 6:**
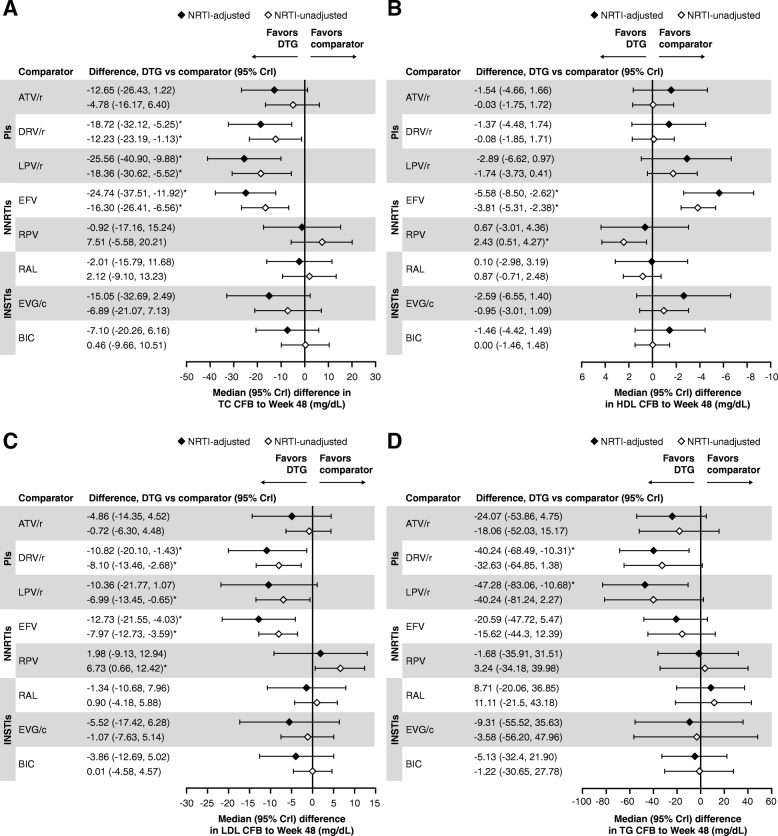
**a** TC, **b** HDL, **c** LDL, and **d** TG CFB with DTG versus comparators. RE model. *Indicates treatment comparisons are significantly different. ATV/r, ritonavir-boosted atazanavir; BIC, bictegravir; CFB, change from baseline; Crl, credible interval; DTG, dolutegravir; DRV/r, ritonavir-boosted darunavir; EFV, efavirenz; EVG/c, cobicistat-boosted elvitegravir; HDL, high-density lipoprotein; INSTIs, integrase strand inhibitors; LDL, low-density lipoprotein; LPV/r, lopinavir-boosted ritonavir; NNRTI, non-nucleoside reverse transcriptase inhibitors; NRTI, nucleoside/nucleotide reverse transcriptase inhibitor; OR, odds ratio; PIs, protease inhibitors; RAL, raltegravir; RE, random effect; RPV, rilpivirine; TC, total cholesterol; TG, triglycerides

Based on model diagnostics [94], the RE model was used for the primary interpretation of safety outcomes. Results of NRTI-adjusted safety analyses with connectors using the RE model are presented below. The analyses of the safety outcomes using NRTI-unadjusted RE models (Figs. 5 and 6) and both NRTI-adjusted and unadjusted FE models (data not shown) were generally consistent with those using the NRTI-adjusted RE models. Similarly, analyses without connector studies (data not shown) yielded generally consistent results to the analysis with connectors presented. Details of the results of the heterogeneity assessments for the safety outcomes can be found in Additional file 1.

The odds of having an AE were significantly lower with DTG compared with LPV/r and were similar to all other core agents (Fig. 5a). The odds of discontinuing treatment for any reason were significantly lower with DTG compared with ritonavir-boosted PIs and NNRTIs, and similar to other INSTIs (Fig. 5b). The odds of discontinuing treatment due to AEs were significantly lower with DTG compared with EFV, ATV/r, and LPV/r, and were similar to all other core agents (Fig. 5c). Increases from baseline in lipid levels were generally similar with DTG and other core agents (Fig. 6a, b, c, and d).

### Subgroup analysis

According to a feasibility analysis, only VS at Week 48 could be analyzed in the predefined subgroups of interest. In total, 18 studies reported VS data in these subgroups, 16 included in the NMA [15, 16, 18, 62–64, 68, 71–75, 78, 79, 87–89, 92], and two included in the subgroup analysis only [92, 93]. Data from three studies that evaluated EFV versus RPV in patients with a baseline VL > 100,000 copies/mL were included in the subgroup analysis [71, 87, 88], despite RPV being indicated only for patients with a VL ≤100,000 copies/mL [95, 96]. Overall, 7093 patients with a baseline VL ≤100,000 RNA copies/mL, 4268 with a baseline VL > 100,000 copies/mL, 5741 with a baseline VL ≤500,000 RNA copies/mL, and 441 with a baseline VL > 500,000 RNA copies/mL, and 1901 and 4823 patients with baseline CD4^+^ ≤ 200 and > 200 cells/μL, respectively, were included. In the two studies that evaluated BIC, patients were classified according to baseline VL ≤400,000 and > 400,000 RNA copies/mL; for the purposes of this analysis these patients were included in the groups with baseline VL ≤500,000 and > 500,000, respectively. VS data were analyzed in the subgroups using NRTI-unadjusted models only, due to the small number of studies and the lack of studies investigating the same core agent with different NRTIs.

**Fig. 7:**
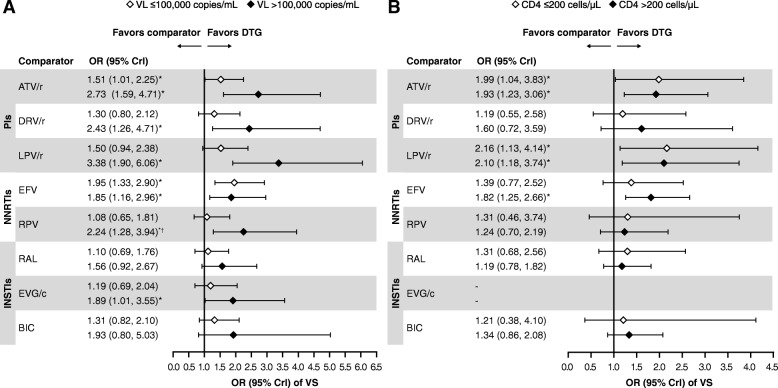
VS at Week 48 by baseline VL (**a**) and CD4^+^ (**b**) with DTG versus comparators. NRTI-unadjusted, FE model. *Indicates treatment comparisons are significantly different. ^†^RPV is not indicated for patients with a VL > 100,000 copies/mL. Studies that reported data on CD4^+^ subgroups used a mix of cutoff inequalities (</≥, ≤/> and </> 200 cells/μL); the convention ≤/> 200 cells/μL used here reflects those used in the majority of studies. ATV/r, ritonavir-boosted atazanavir; BIC, bictegravir; Crl, credible interval; DTG, dolutegravir; DRV/r, ritonavir-boosted darunavir; EFV, efavirenz; EVG/c, cobicistat-boosted elvitegravir; FE, fixed effect; INSTIs, Integrase strand inhibitors; LPV/r, lopinavir-boosted ritonavir; NNRTIs, non-nucleoside reverse transcriptase inhibitors; NRTI, nucleoside/nucleotide reverse transcriptase inhibitor; OR, odds ratio; PIs, protease inhibitors; RAL, raltegravir; RPV, rilpivirine; VL, viral load; VS, virologic suppression

The odds of achieving VS at Week 48 in patients with baseline VL > 100,000 RNA copies/mL were statistically superior with DTG compared with all core agents except RAL and BIC (Fig. 7a). There was a higher probability of patients with baseline VL > 100,000 RNA copies/mL achieving VS at Week 48 with DTG versus all other core agents (93–100%). The ORs for VS at Week 48 in patients with baseline VL > 500,000 RNA copies/mL are shown in Additional file 1: Figure S1.

The odds of achieving VS at Week 48 in patients with ≤200 CD4^+^ cells/μL at baseline were statistically superior with DTG compared with ATV/r and LPV/r, and similar to all other core agents analyzed for this outcome (Fig. 7b). In this subgroup, DTG was associated with a higher probability of more patients achieving VS at Week 48 versus other core agents (62–99%).

## Discussion

DTG is among the most effective core agents available for the initial treatment of patients with HIV-1 infection, according to the results of this NMA. In treatment-naïve patients, the odds of achieving VS at Week 48 were significantly higher with DTG than all ritonavir-boosted PIs and NNRTIs, and numerically higher than other INSTIs, although not significantly so, after adjustment for the choice of NRTI. These results were consistent with those of previous NMAs [11, 12]. Furthermore, DTG was more likely to result in patients achieving VS at Week 48 versus all other core agents, including ritonavir-boosted PIs (100%), NNRTIs (99–100%), and other INSTIs (94–97%) (NNRTI-adjusted, FE model). The change in CD4^+^ cell count from baseline to Week 48 in patients receiving DTG was also significantly higher or similar to that with other core agents. The benefits of DTG were achieved without any additional risk, such as AEs, discontinuations, discontinuations due to AEs, and changes in lipid levels compared with other core agents. Generally consistent results were observed in all models used (NRTI-adjusted and -unadjusted, FE and RE, with and without connector studies). These data suggest that INSTIs are a superior core agent class, and that DTG is among the most effective core agents available.

The results of this NMA were consistent in subgroups of patients with high VL or low CD4^+^ cell counts at baseline, who can be difficult to treat. In patients with VL > 100,000 RNA copies/mL at baseline, the odds of achieving VS at Week 48 were statistically superior with DTG compared with all core agents except RAL and BIC, and DTG was associated with a 93–100% probability of patients achieving VS at Week 48 versus all other core agents, including RAL and BIC, in this subgroup. Results in patients with VL > 500,000 RNA copies/mL at baseline were generally consistent with these findings, although highly variable due to the low number of patients in this subgroup. The subgroup with a VL > 500,000 RNA copies/mL also included studies for which data were reported at a different threshold (> 400,000 RNA copies/mL in the two studies evaluating BIC). In patients with CD4^+^ ≤ 200 cells/μL at baseline, the odds of achieving VS at Week 48 were statistically superior with DTG compared with ATV/r and LPV/r, and similar to all other core agents. DTG was associated with a 62–99% probability of patients achieving VS at Week 48 versus all other core agents in this subgroup. These results support the continued use of DTG as a preferred core agent, including in resource-constrained settings; indeed, by the end of 2017, approximately 70 lower and middle-income countries had already included/were planning to include DTG in their national formularies and to shift to a DTG-based first-line regimen [10].

Given the cost and complications associated with conducting further RCTs to directly compare the increasing number of core agents available for the treatment of HIV-1 infection, it is appropriate to use robust methods such as an NMA to synthesize the available evidence for new and established agents in a single analysis [97]*.* The NMA methods used here were generally consistent with those of previous studies [11, 12], with the addition of probabilistic results to rank therapies. Unlike previous NMAs, which did not include data for the NRTI TAF as it was not recommended at the time, this NMA included grouped data on TDF or TAF in combination with core agents. The grouping of TDF and TAF could be perceived as a limitation of this analysis, due to the possibility of these NRTIs having different effects independent of the core agent. However, data from head-to-head studies in which TAF and TDF (both with EVG/c and FTC) were compared in treatment-naïve patients with HIV-1 support this approach, as TAF was shown to be non-inferior to TDF in terms of VS, with similar safety profiles [34]. No previous NMA has included BIC, as they were undertaken before its approval in 2018 [11, 12]. The US DHHS and EACS now recommend the INSTIs BIC in addition to DTG and RAL as preferred first-line core agents for treatment-naïve adults, while the WHO does not recommend BIC or RAL, recommending a DTG-based regimen [7, 8, 10]. The current analyses included all recently published studies evaluating core agents for treatment-naïve patients with HIV, including BIC, and allowed them to be ranked based on their ability to achieve VS relative to DTG*.* Overall, the results of this analysis are in line with those of previous NMAs, with INSTIs having superior efficacy to ritonavir-boosted PIs and NNRTIs in treatment-naïve patients [11, 12]*.* The 2016 NMA by Kanters et al found a clear hierarchy within the INSTI class with regard to their ability to achieve VS, with DTG being the most efficacious followed by RAL, then EVG/c [11]. The VS results at Week 48 from the current analysis are very similar to those reported by Kanters et al, with DTG being the most efficacious followed by RAL, BIC, then EVG/c.

## Conclusions

In conclusion, our systematic literature review and NMA provide further evidence to support INSTIs as the superior class of core agent for first-line treatment of HIV-1 infection in treatment-naïve patients. They further suggest that DTG is among the most effective first-line core agents, with a safety profile similar to other core agents at Week 48. In NRTI-adjusted models in treatment-naïve patients with HIV-1, the odds of achieving VS at Week 48 were significantly higher with DTG than with all ritonavir-boosted PIs and NNRTIs and similar to other INSTIs, and increases in CD4^+^ cell count with DTG were significantly higher than with all ritonavir-boosted PIs and EFV and similar to other core agents. Higher odds of achieving VS at Week 48 were also seen with DTG compared with all other core agents in patients with VL > 100,000 RNA copies/mL or CD4^+^ cell counts ≤200 cells/μL at baseline, who can be difficult to treat. Overall, the results of this NMA confirm that DTG should remain a preferred core agent in treatment-naïve patients infected with HIV-1.

## Additional file

Additional file 1: Contains additional study methods, search terms, summary of NMA inputs, EPHPP quality assessment ratings, GRADE assessments, median change in VS at Week 48 by VL at baseline [≤ or > 500,000 RNA copies/mL]). (DOCX 321 kb)

## Abbreviations

3TC: Lamivudine
ABC: Abacavir
AE: Adverse event
AIDS: Acquired immunodeficiency syndrome
ATV: Atazanavir
ATV/r: Ritonavir-boosted atazanavir
BIC: Bictegravir
CADTH: Canadian Agency for Drugs and Technologies in Health
cART: Combination antiretroviral therapy
CDC: Centers for Disease Control and Prevention
CFB: Change from baseline
CrI: Credible interval
CSR: Clinical study report
CVR: Confirmed virologic response
DHHS: Department of Health and Human Services
DIC: Deviance Information Criterion
DRV/r: Ritonavir-boosted darunavir
DTG: Dolutegravir
EACS: European AIDS Clinical Society
EFV: Efavirenz
EMA: European Medicines Agency
EPAR: European Public Assessment Report
EPHPP: Effective Public Health Practice Project Quality Assessment
EVG/c: Cobicistat–boosted elvitegravir
FDA: Food and Drug Administration
FE: Fixed effect
FPV/r: Ritonavir-boosted fosamprenavir
FTC: Emtricitabine
GRADE: Grading of Recommendations Assessment, Development and Evaluation
HDL: High-density lipoprotein
HIV-1: Human immunodeficiency virus type 1
INSTIs: Integrase strand inhibitors
LDL: Low-density lipoprotein
LPV/r: Ritonavir-boosted lopinavir
NCT: National Institute of Health clinical trial
NFV: Nelfinavir
NICE: National Institute for Health and Care Excellence
NMA: Network meta-analysis
NNRTIs: Non-nucleoside reverse transcriptase inhibitor
NRTI: Nucleoside/nucleotide reverse transcriptase inhibitors
NVP: Nevirapine
OR: Odds ratio
PI: Protease inhibitor
PRISMA: Preferred Reporting Items for Systematic Reviews and Meta-Analyses
RAL: Raltegravir
RCT: Randomized controlled trial
RE: Random effect
RPV: Rilpivirine
SLR: Systematic literature review
SQV/r: Ritonavir-boosted saquinavir
TAF: Tenofovir alafenamide fumarate
TC: Total cholesterol
TDF: Tenofovir disoproxil fumarate
TE: Treatment experienced
TG: Triglycerides
TLOVR: Time to loss of virologic response
TN: Treatment naïve
VL: Viral load
VS: Virologic suppression
WHO: World Health Organization
