# Supplementary material for: Comparative efficacy and safety of dolutegravir relative to common core agents in treatment-naïve patients infected with HIV-1: a systematic review and network meta-analysis
Source: BMC Infect Dis. 2019 May 30;19:484. doi: 10.1186/s12879-019-3975-6 (PMC6543679; doi:10.1186/s12879-019-3975-6)
Supplement: Supplementary file 1 — Contains additional study methods, search terms, summary of NMA inputs, EPHPP quality assessment ratings, GRADE assessments, median change in VS at Week 48 by VL at baseline [≤ or > 500,000 RNA copies/mL]). (DOCX 321 kb) [file 12879_2019_3975_MOESM1_ESM.docx]

**Supplementary materials, Additional File 1**

**Methods**

***Grading of Recommendations Assessment (GRADE) quality of evidence (as used in the current analysis)***

The GRADE algorithm uses a step-by-step approach to rate the quality of comparisons of treatment effects as High, Moderate, Low, or Very Low.

**Step 1** Present direct effect estimates from head-to-head studies and indirect estimates from connector studies (two or more head-to-head studies that share a common comparator i.e. not a core agent of interest).

**Step 2** Rate the quality of each direct effect estimate. Randomized controlled trials all start as high quality. Rates are then adjusted down by 1 grade (Serious Concern) or down by 2 grades (Very Serious Concerns) for the following reasons:

- Inconsistency (or heterogeneity in the context of network meta-analyses [NMA]) ‒ degree to which the effect is dissimilar or inconsistent with others of the same connection.
- Indirectness – generalizability of the study participants to the target population of interest.
- Imprecision – effects with wide confidence intervals or null effect (no study was downgraded for insignificant study effect sizes as many had non-inferiority designs; studies with small sample sizes were downgraded).
- Risk of bias – study quality with respect to blinding and allocation of treatment (open-label studies were not downgraded, as HIV studies are often open label).
- Publication bias – evidence that smaller, negative trials of a specific comparison may not be published (due to the large number of comparisons in our network, there was no detection of publication bias).

**Step 3** Rate the quality of each indirect effect estimate based on the ratings of the comparisons forming the indirect evidence loop.

**Step 4** Rate the quality of the NMA estimate. Direct evidence rating was given preference over indirect evidence when differences occurred.

The GRADE quality tables have been previously published (1).

***Analysis of heterogeneity of treatment effects***

Heterogeneity of treatment effects were assessed by the Q statistic, where a p-value >0.1 was considered to demonstrate significant heterogeneity for pairs of treatment comparisons with two or more studies. The I^2^ statistic, calculated based on the Q statistic, was used to assess the proportion of variability attributable to heterogenicity; an I^2^ value <30% is generally considered as mild heterogeneity while >50% indicates the presence of substantial heterogeneity (2). In addition, a network inconsistency model (3) was used to evaluate the inconsistency of the virologic suppression (VS) and CD4^+^ cell count outcomes including connectors by constructing a meta-analysis estimating only direct comparison evidence without the influence of the network or indirect treatment effects.

# Results

***Heterogeneity assessments: efficacy outcomes***

**Viral suppression**

Five direct comparisons were informed by at least two studies. Heterogeneity in the odds ratio (OR) for VS was observed for the comparison between efavirenz (EFV) and ritonavir-boosted atazanavir (ATV/r) reported in the Altair (4) and NORTHIV (5) studies (Q statistics p=0.0956; I^2^=64%). In these two studies, the estimated OR for VS between the treatments was found to be in opposite directions (0.73 and 1.85, respectively). The NORTHIV study included patients with lower median CD4^+^ cell counts at baseline (~150 cells/μL vs ~225 cells/μL) and more patients with Centers for Disease Control and Prevention (CDC) stage C disease, i.e. the most severe phase of infection, (22% vs 5%). However, it is unclear if these differences in patient characteristics would result in differential treatment effects. In addition, these studies were relatively small, with less than 115 patients per arm, so the precision of the study results was low. Given that both studies met the study inclusion criteria and both represent part of the target population, including these studies in the NMA contributes to the body of evidence and the generalizability of the results. No substantial inconsistency was identified between NMA and direct evidence estimates of VS.

**CD4+ cell count**

Two direct comparisons were informed by at least two studies. No heterogeneity was observed in the mean difference in CD4^+^ cell count change from baseline for either of the two direct comparisons. Inconsistency was identified between NMA and direct evidence estimates of change from baseline in CD4^+^ cell count in the Lake study (6), which has been previously reported (7).

***Heterogeneity assessments: safety outcomes***

Adverse events (AEs)

Three direct comparisons were informed by at least two studies. Heterogeneity in the ORs for AEs for each comparison was identified for ATV/r versus ritonavir-boosted lopinavir (LPV/r), reported by the CASTLE and NORTHIV studies (5, 8). In both of these studies there was a higher probability of AEs with LPV/r than ATV/r, but the NORTHIV study effect was larger. The NORTHIV study included patients with lower median CD4^+^ cells at baseline (150–170 vs 204–205), more patients with CDC stage C disease (18%–22% vs 5%), and was a small study (77–81 vs 440–443 patients per arm).

Heterogeneity was also identified between the two bictegravir (BIC) versus dolutegravir (DTG) studies. This could reflect differences in the two methods of capturing AEs (9, 10). The 2017 study reported by Gallant *et al*. reported the number of patients with at least one AE in the trial, whereas the clinicaltrials.gov study reported the number of patients who experienced at least one of the AEs occurring with ≥5% frequency.

Discontinuations

Three direct comparisons were informed by at least two studies. No significant heterogeneity was observed in the ORs of discontinuation for any comparison.

Discontinuation due to AE

Four direct comparisons were informed by at least two studies. Heterogeneity was identified in the ORs of discontinuation due to AEs for EFV versus LPV/r.

Supplementary Table 1: Search terms used to identify relevant citations from PubMed and Embase

|  | **PubMed** | **Embase** |
| --- | --- | --- |
| **General search terms** | HIV-1 [mesh] OR HIV infections [mesh]) NOT pregnancy [mesh] | ('human immunodeficiency virus 1'/exp OR 'human immunodeficiency virus infection'/exp NOT 'pregnancy'/exp) |
| **Treatment-specific search terms** | ((dolutegravir OR GSK1349572) OR (efavirenz OR Sustiva OR Stocrin OR DMP-266) OR (raltegravir OR Isentress OR MK-0518) OR (elvitegravir OR GS-9137 OR JTK-303) OR (rilpivirine OR Edurant OR TMC 278) OR (darunavir OR Prezista OR TMC-114) OR (atazanavir OR Reyataz OR BMS-232632) OR (lopinavir OR ABT-378 OR Aluviran OR Kaletra) OR (etravirine OR Intelence OR TMC-125) OR Atripla OR Quad OR Stribild OR Eviplera OR Complera OR bictegravir OR GS-9883)) | 'dolutegravir'/exp OR 'efavirenz'/exp OR 'raltegravir'/exp OR 'elvitegravir'/exp OR 'rilpivirine'/exp OR 'darunavir'/exp OR 'darunavir plus ritonavir'/exp OR 'atazanavir'/exp OR 'atazanavir plus ritonavir'/exp OR 'lopinavir'/exp OR 'lopinavir plus ritonavir'/exp OR 'etravirine'/exp OR 'efavirenz plus emtricitabine plus tenofovir disoproxil'/exp OR 'emtricitabine plus rilpivirine plus tenofovir disoproxil'/exp OR 'cobicistat'/exp OR ‘bictegravir’/exp |
| **Limits** | Humans, Randomized Controlled Trial, English, Adults and Adolescents (≥13 years of age), systematic reviews, meta-analyses | |

HIV, human immunodeficiency virus.

**Supplementary Table 2: Summary of NMA inputs for the 36 studies in treatment-naïve patients included in the NMA and the two additional studies included in the subgroup analysis of VS only**

| **Study** | **Core agent** | **N** | **% male** | **Age, y** | **Baseline CD4^+^, cells/mL (SD)** | **Baseline viral load, log_10_ RNA copies/mL, (SD)** | **CD4+ change, cells/µL (SD)** | **VS HIV RNA <50 copies/mL (n/N)** | **AEs (n/N)** | **Discontinuations (n/N)** | **Discontinu ations due to AEs (n/N)** | **TC change, mg/dL (SD)** | **HDL change, mg/dL (SD)** | **LDL change, mg/dL (SD)** | **TG change, mg/dL (SD)** |
| --- | --- | --- | --- | --- | --- | --- | --- | --- | --- | --- | --- | --- | --- | --- | --- |
| 089 Study  (11, 12) | ATV/r | 95 | 72.63 | 35 | 201 | 4.8 | 174 | 71/95 | - | 11/95 | 8/95 | 24 | 9 | 22 | 14 |
|  | ATV | 105 | 70.48 | 34 | 194 | 5.1 | 213 | 73/105 | - | 10/105 | 1/105 | 11 | 9 | 16 | -15 |
| 2NN  (13, 14) | EFV | 400 | 63.50 | 34.7 | 190 | 4.7 | 160 | 280/400 | 72/400 | 44/381 | - | - | - | - | - |
|  | NVP | 387 | 60.98 | 33.9 | 170 | 4.7 | 160 | 253/387 | 79/387 | 56/378 | - | - | - | - | - |
| 934 Study  (15-17) | EFV | 254 | 87.01 | 38 | 245 (156.6) | 5 (0.51) | 158 (111.7) | 173/254 | - | - | - | 35 | 9 | 20 | 31 |
|  | EFV | 255 | 85.88 | 38 | 246 (171.9) | 5.03 (0.54) | 190 (107.3) | 196/255 | - | - | - | 21 | 6 | 13 | 3 |
| ACTG A5142 (18, 19) | EFV | 212† | 81.20 | 39 | 195 | 4.8 | - | 177/212 | - | - | - | - | - | - | - |
|  | LPV/r | 217† | 76.68 | 37 | 190 | 4.8 | - | 165/217 | - | - | - | - | - | - | - |
| ACTG A5202 (15, 20) Study arms: ATV and EFV each with ABC/3TC & TDF/FTC | ATV/r | 463 | 83.80 | 38 | 236 | 4.7 (0.7) | 178 | - | - | - | - | 29 | 8 | 13 | 24 |
|  | ATV/r | 465 | 83.23 | 38.9 | 224 | 4.7 (0.7) | 175 | - | - | - | - | 10 | 4.8 | 2 | 14 |
|  | EFV | 465 | 78.92 | 38.4 | 225 | 4.7 (0.7) | 188 | - | - | - | - | 40 | 12 | 20.5 | 15 |
|  | EFV | 464 | 84.70 | 38.2 | 234 | 4.7 (0.7) | 163 | - | - | - | - | 22 | 8 | 10 | 13 |
| ALERT (21) | ATV/r | 53 | 88.68 | 40 | 188 | 4.890 | 183 | 44/53 | - | 4/53 | 1/53 | 10 | 14 | -6 | - |
|  | FPV/r | 53 | 79.25 | 40 | 161 | 4.924 | 170 | 40/53 | - | 8/53 | 1/53 | 13 | 11 | 2 | - |
| Altair  (4) | EFV | 114 | 78.95 | 37.3 | 227 (95) | 4.67 (0.63) | 187 | 97/114 | 99/114 | 7/114 | - | - | - | - | - |
|  | ATV/r | 105 | 71.43 | 36.7 | 235 (114) | 4.77 (0.58) | 192 | 93/105 | 95/105 | 6/105 | - | - | - | - | - |
| ARIA  (22) | DTG | 248 | 0.00 | 37.5 | 340 | 4.41 | 234 | 203/248 | 195/248 | 42/248 | 10/248 | - | - | - | - |
|  | ATV/r | 247 | 0.00 | 37 | 350 | 4.43 | 200 | 176/247 | 197/247 | 55/247 | 18/247 | - | - | - | - |
| ARTEMIS (23-25) | DRV/r | 343 | 69.65 | 35.5 | 228 | 4.86 (0.64) | 137 | 288/343 | 309/343 | 41/343 | 12/343 | 22 | 5 | 12 | 23 |
|  | LPV/r | 346 | 69.68 | 35.3 | 218 | 4.84 | 141 | 270/346 | 328/346 | 56/346 | 24/346 | 31 | 7 | 12 | 9 |
| ASSERT (26, 27) | EFV | 193 | 79.79 | 36 | 230 | 5.12 | 150 | 137/193 | - | 44/193 | 20/193 | - | - | - | - |
|  | EFV | 192 | 82.81 | 38 | 240 | 5.01 | 150 | 114/192 | - | 63/192 | 25/192 | - | - | - | - |
| ATADAR  (28) | ATV/r | 90 | 86.67 | 35 | 328 (205) | 4.8 (0.7) | - | - | - | - | - | 9.64 (34.05) | 5.2 (10.42) | -2.88 (25.36) | 41.04 (81.97) |
|  | DRV/r | 88 | 88.64 | 37 | 341 (171) | 4.8 (0.8) | - | - | - | - | - | 11.37 (28.17) | 4.67 (4.67) | 4.89 (26.95) | 17.13 (63.46) |
| BASIC (29) | ATV/r | 61 | 86.89 | 38 | 249 (116) | 4.8 (0.7) | 161 (124) | 48/61 | - | 8/61 | - | 8.88 (33.45) | 4.63 (8.51) | - | 1.77 |
|  | SQV/r | 57 | 82.46 | 38.8 | 234 (102) | 4.7 (0.7) | 190 (111) | 43/57 | - | 7/57 | - | 9.65 (31.54) | 8.88 (12.26) | - | 1.77 |
| CASTLE (8, 30-32) | ATV/r | 440 | 68.64 | 34 | 205 | 5.01 | 203 | 343/440 | 400/441 | 39/438 | 10/438 | 17 | 9 | 11 | 14 |
|  | LPV/r | 443 | 68.62 | 36 | 204 | 4.96 | 219 | 338/443 | 399/437 | 58/440 | 14/440 | 38 | 11 | 17 | 58 |
| CLASS  (33) | EFV | 97 | 82.47 | 36.6 | 307 (184) | 4.9 (0.67) | 194 | 73/97 | - | - | - | - | - | - | - |
|  | FPV/r | 96 | 86.46 | 36.2 | 306 (192) | 4.85 (0.52) | 167 | 57/96 | - | - | - | - | - | - | - |
| Clumeck 2014 (34) | LPV/r | 216 | 27.31 | 38 | 168 | 5.13 | 125 | 145/216 | 59/216** | 56/216 | - | - | - | - | - |
|  | NVP | 209 | 29.19 | 38 | 164 | 5.17 | 119 | 142/209 | 34/209** | 54/209 | - | - | - | - | - |
| CNA30024 (35) | EFV | 324 | 79.63 | 35 | 267 | 4.81 | 209 | 226/324 | 226/324 | 49/327 | - | - | - | - | - |
|  | EFV | 325 | 82.15 | 35 | 258 | 4.76 | 155 | 224/325 | 250/325 | 57/327 | - | - | - | - | - |
| ECHO (30, 36, 37) | EFV | 344 | 79.94 | 36 | 257 | 5 | 182 | 285/344 | 317/344 | 56/344 | 27/344 | 24.3621 (36.5362) | 9.266409 (10.961) | 11.96911 (29.229) | 14.15929 |
|  | RPV | 346 | 77.46 | 36 | 240 | 5 | 196 | 287/346 | 303/346 | 50/346 | 8/346 | 1.1601 (29.31381) | 2.702703 (10.993) | -1.5444 (25.6496) | -8.84956 |
| FLAMINGO (38)  VS data for 4 arms: DTG & DRV/r, each with ABC/3TC and TDF/FTC | DRV/r | 242 | 83.06 | 34 | 400 | 4.48 | 215.4 (177.26) | 68/80 | 205/242 | 29/242 | 9/242 | 25.5222 | 2.7069 | 14.6946 | 41.6279 |
|  | DRV/r |  |  |  |  |  |  | 132/162 |  |  |  |  |  |  |  |
|  | DTG | 242 | 87.19 | 34 | 390 | 4.49 | 243.8 (180.68) | 71/79 | 206/242 | 18/242 | 3/242 | 3.4803 | 1.5468 | 2.3202 | -3.5428 |
|  | DTG |  |  |  |  |  |  | 146/163 |  |  |  |  |  |  |  |
| GS-236-0102 (QUAD) (39-41) | EFV | 352 | 89.77 | 38 | 382 (170.2) | 4.78 (0.6) | 206 (153.4) | 296/352 | 334/352 | 46/352 | 18/352 | 18.9483 | 7.734 | 16.98842 | - |
|  | EVG/c | 348 | 88.22 | 38 | 391 (188.6) | 4.73 (0.6) | 239 (167.2) | 305/348 | 650/701 | 37/348 | 12/348 | 9.6675 | 5.0271 | 10.03861 | - |
| GS-236-0103 (QUAD) (42, 43) | EVG/c | 353 | 91.78 | 38 | 351 | 4.8 (0.61) | 207 (164.2) | 316/353 |  | 33/353 | 13/353 | 10.0542 | 5.8005 | 10.81081 | 7.97 |
|  | ATV/r | 355 | 89.01 | 39 | 366 | 4.8 (0.62) | 211 (160.3) | 308/355 | 333/355 | 40/355 | 18/355 | 8.1207 | 5.0271 | 10.42471 | 23.01 |
| GS-US-380-1489 (9) | BIC | 314 | 90.76 | 31 | 443 | 4.42 | 233 (185.2) | 290/314 | 265/314 | 19/314 | 0/314 | 13 | 5 | 7 | 9 |
|  | DTG | 315 | 89.52 | 32 | 450 | 4.51 | 229 (188.8) | 293/315 | 283/315 | 16/315 | 4/315 | 11 | 5 | 4 | 3 |
| GS-US-380-1490 (10, 44) | BIC | 320 | 87.50 | 33 | 440 | 4.43 | 180 (166.6) | 286/320 | 264/320* | 28/320 | 5/320 | 12 | 5 | 9 | 3 |
|  | DTG | 325 | 88.62 | 34 | 441 | 4.45 | 201 (166.4) | 302/325 | 272/325* | 20/325 | 1/325 | 15 | 5 | 12 | 7 |
| HEAT (39, 45) | LPV/r | 345 | 80.00 | 38 | 193 | 4.844 | 173 | 210/345 | - | - | - | - | - | - | - |
|  | LPV/r | 343 | 83.97 | 38 | 214 | 4.903 | 201 | 216/343 | - | - | - | - | - | - | - |
| IMEA 040 (46)^‡^ | ATV/r | 59 | 79.66 | 45 | 75 | 5.4 | - | 39/59* | - | - | - | - | - | - | - |
|  | DRV/r | 61 | 73.77 | 42 | 55 | 5.45 | - | 49/61* | - | - | - | - | - | - | - |
| INITIO (47) | EFV | 297 | 78.11 | 38.8 | 221 (183) | 4.9 (0.78) | 160 | - | - | - | - | - | - | - | - |
|  | NFV | 311 | 79.42 | 38.5 | 223 (168) | 4.95 (0.67) | 162 | - | - | - | - | - | - | - | - |
| KLEAN (48, 49) | LPV/r | 444 | 78.38 | 37 | 194 | 5.1 | 191 | 288/444 | - | 97/444 | 24/444 | 53 | 14 | 23 | - |
|  | FPV/r | 434 | 77.88 | 38 | 188 | 5.1 | 176 | 285/434 | - | 93/434 | 23/434 | 61 | 13 | 28 | - |
| Lake (6) | EFV | 63 | 85.71 | 39 | 193 (122) | 5.4 | 298 | 36/63 | - | 18/63 | 14/63 | 48 (32.08) | - | - | - |
|  | LPV/r | 63 | 87.30 | 37 | 191 (127) | 5.3 | 249 | 40/63 | - | 23/63 | 8/63 | 44 (40.75) | - | - | - |
| M98-863 (50) | LPV/r | 326 | 79.75 | 38.4 | 260 (214) | 4.89 (0.75) | 207 | - | - | - | - | - | - | - | - |
|  | NFV | 327 | 80.74 | 37.3 | 258 (196) | 4.92 (0.74) | 195 | - | - | - | - | - | - | - | - |
| METABOLIK (51, 52) | ATV/r | 31 | 87.10 | 35 | 316 | 4.6 (0.7) | 205.3 | - | 29/31 | 6/31 | 2/31 | 11.8 (31.9) | 3.7 (9.9) | 13.9 (27.1) | 9.6 (73.7) |
|  | DRV/r | 34 | 85.29 | 36.5 | 267 | 5 (0.8) | 217.4 | - | 31/34 | 5/34 | 0/34 | 22.3 (30.7) | 6 (7.4) | 14.7 (25.9) | 26.1 (69) |
| Montaner 2006 (53) | EFV | 77 | 75.32 | 37.2 | 343 (180) | 4.7 (0.5) | 204 | 55/77 | - | 23/80 | - | 42 | 12.1 | - | 71.45 |
|  | SQV/r | 75 | 70.67 | 37.2 | 372 (190) | 4.8 (0.6) | 239 | 38/75 | - | 28/81 | - | 32 | 8.7 | - | 51.2 |
| NORTHIV (5)  2013) | EFV | 77 | 53.25 | 37 | 150 | 5.3 | - | 62/77 | 4/77 | 5/77 | 5/77 | - | - | - | - |
|  | ATV/r | 81 | 61.73 | 39 | 170 | 5.17 | - | 56/81 | 2/81 | 6/81 | 6/81 | - | - | - | - |
|  | LPV/r | 81 | 55.55 | 37 | 150 | 5.34 | - | 54/81 | 2/81 | 12/81 | 6/81 | - | - | - | - |
| Sierra-Madero 2010 (54)^‡^ | EFV | 95 | 83.16 | 36.7 | 64 | - | - | 67/95* | - | - | - | - | - | - | - |
|  | LPV/r | 94 | 87.23 | 36 | 52 | - | - | 50/94* | - | - | - | - | - | - | - |
| SINGLE (55) | DTG | 414 | 83.82 | 36 | 334.5 | 4.67 | 267.06 (184.22) | 364/414 | 369/414 | 51/414 | 10/414 | 17.0148 (35.58) | 5.22 (8.68) | 8.494208 (24.9421) | 17.66 (94.37) |
|  | EFV | 419 | 84.96 | 35 | 339 | 4.7 | 208.16 (190.65) | 338/419 | 387/419 | 84/419 | 42/419 | 23.9754 (39.06) | 7.95 (11.02) | 13.12741 (24.8798) | 18.61 (91.83) |
| SPRING-2 (56) | DTG | 411 | 84.67 | 37 | 359 | 4.52 | 230 | 145/169 | 339/411 | 47/411 | 8/411 | 6.9606 (27.7992) | 2.7069 (10.811) | 3.088803 (21.6216) | 8.849558 (91.15044) |
|  | DTG |  |  |  |  |  |  | 216/242 |  |  |  |  |  |  |  |
|  | RAL | 411 | 86.37 | 35 | 362 | 4.58 | 230 | 142/164 | 339/411 | 56/411 | 6/411 | 8.8941 (29.34363) | 2.7069 (10.425) | 3.474903 (22.7799) | 8.849558 (92.0354) |
|  | RAL |  |  |  |  |  |  | 209/247 |  |  |  |  |  |  |  |
| Squires 2004 (13) Jemsek 2006 (lipid analysis) (57) | EFV | 401 | 66.08 | 33 | 280 | 4.91 | 160 | 150/401 | - | 79/401 | 34/401 | - | - | - | - |
|  | ATV | 404 | 63.61 | 33 | 286 | 4.87 | 176 | 131/404 | - | 65/404 | 26/404 | - | - | - | - |
|  | ATV | 111 | 73.87 | 30 | 328 | 4.84 | - | - | - | - | - | 0 | 3 | -2 | -12 |
|  | EFV | 100 | 71.00 | 29 | 323 | 4.69 | - | - | - | - | - | 31 | 7 | 17 | 15 |
| STaR (GS-US-264-0110) (58, 59) | EFV | 392 | 92.86 | 35 | 385 (187) | 4.8 (0.6) | 191 (144) | 320/392 | - | 72/392 | 34/392 | 22 | 8 | 14 | 8 |
|  | RPV | 394 | 92.89% | 37 | 396 (180) | 4.8 (0.7) | 200 (159) | 338/394 | - | 54/394 | 10/394 | 1 | 2 | 1 | -8 |
| STARMRK (60-63) | EFV | 282 | 81.91% | 36.9 | 217.4 (133.6) | 5 (0.6) | 163.3 (121.2) | 230/281 | 272/282 | 35/282 | 17/282 | 32.87 (33.64) | 10.05 (10.83) | 16.24 (29) | 37.2 |
|  | RAL | 281 | 80.78% | 37.6 | 218.9 (124.2) | 5.0 (0.6) | 189.1 (122.9) | 241/280 | 253/281 | 24/281 | 8/281 | 10.05 (29) | 4.25 (8.51) | 5.8 (24.75) | -2.66 |
| THRIVE (52, 64) | EFV | 338 | 72.19% | 36.3 | 263 | 5 | 171 (150.1) | 276/338 | 312/338 | 56/338 | 25/338 | 30.5493 | 10.42 | 16.98842 | 12.3998 |
|  | RPV | 340 | 73.53% | 36.3 | 263 | 5 | 189 (136.2) | 291/340 | 313/340 | 44/340 | 15/340 | 3.0936 | 4.25 | -0.7722 | -6.1999 |

^†^Number of patients at risk at Week 48; ^‡^studies included in the subgroup analyses of VS only.

3TC, Lamivudine; ABC, Abacavir; AE, adverse event; ATV, atazanavir; ATV/r, ritonavir-boosted atazanavir; BIC, bictegravir; DTG, dolutegravir; DRV, darunavir; DRV/r, ritonavir-boosted darunavir; EFV, efavirenz; EVG/c, cobicistat-boosted elvitegravir; HDL, high-density lipoprotein; HIV, human immunodeficiency virus; FPV/r, ritonavir-boosted fosamprenavir; FTC, emtricitabine; LDL, low-density lipoprotein; LPV/r, ritonavir-boosted lopinavir; NFV, nelfinavir; NMA, network meta-analysis; NNRTI, non-nucleoside reverse transcriptase inhibitor; NRTI, nucleotide reverse transcriptase inhibitor; NVP, nevirapine; PI, protease inhibitor; RAL, raltegravir; RNA, ribonucleic acid; RPV, rilpivirine; SD, standard deviation; SQV/r, ritonavir-boosted saquinavir; TC, total cholesterol; TDF, tenofovir disoproxil fumarate; TG, triglycerides; ULN, upper limit of normal; VL, viral load; VS, virologic suppression; y, year.

**Supplementary Table 3: EPHPP quality assessment ratings (1: strong; 2: moderate; 3: weak) for the 36 studies included in the NMA and two additional studies included in the subgroup analyses only (46, 54)**

| Study (Source) | Selection bias | Study design | Confounders | Blinding | Data collection method | Withdrawals and dropout | Global rating |
| --- | --- | --- | --- | --- | --- | --- | --- |
| 089 Study* (12) | 1 | 1 | 1 | 3 | 1 | 1 | **2** |
| 2NN (13) | 1 | 1 | 1 | 1 | 1 | 1 | 1 |
| 934 study (16) | 1 | 1 | 1 | 3 | 1 | 1 | **2** |
| ACTG A5142 (19) | 1 | 2 | 1 | 3 | 1 | 2 | **2** |
| ACTG A5202 (20) | 2 | 1 | 1 | 3 | 1 | 2 | **2** |
| ALERT* (21) | 1 | 2 | 1 | 3 | 1 | 1 | **2** |
| ALTAIR (4) | 1 | 1 | 1 | 3 | 1 | 1 | **2** |
| ARIA (22) | 1 | 1 | 1 | 3 | 1 | 1 | **2** |
| ARTEMIS (25) | 1 | 1 | 1 | 3 | 1 | 1 | **2** |
| ASSERT (27) | 1 | 1 | 1 | 3 | 1 | 2 | **2** |
| ATADAR (28) | 1 | 1 | 2 | 3 | 1 | 1 | **2** |
| BASIC Study* (29) | 2 | 1 | 1 | 3 | 1 | 1 | **2** |
| CASTLE (8) | 1 | 1 | 1 | 3 | 1 | 1 | **2** |
| CLASS* (33) | 2 | 1 | 1 | 3 | 1 | 2 | **2** |
| Clumeck 2014* (34) | 3 | 1 | 1 | 3 | 1 | 2 | 3 |
| CNA30024 (35) | 1 | 2 | 1 | 1 | 1 | 2 | **1** |
| ECHO (36) | 1 | 1 | 1 | 1 | 1 | 1 | **1** |
| FLAMINGO (38) | 1 | 1 | 1 | 3 | 1 | 1 | **2** |
| GS-236-0102 (40) | 1 | 1 | 1 | 1 | 1 | 1 | **1** |
| GS-236-0103 (42) | 1 | 1 | 1 | 1 | 1 | 1 | **1** |
| GS-US-380-1489 (9) | 1 | 1 | 1 | 1 | 1 | 1 | **1** |
| GS-US-380-1490 (10) | 1 | 1 | 1 | 1 | 1 | 1 | **1** |
| HEAT (45) | 1 | 2 | 1 | 1 | 1 | 1 | **1** |
| IMEA 040 DATA*^†^ (46) | 1^‡^ | 2 | 1 | 3 | 1 | 2 | 2 |
| INITIO Trial* (47) | 2 | 1 | 1 | 3 | 1 | 1 | **2** |
| KLEAN* (48) | 1 | 1 | 1 | 3 | 1 | 2 | **2** |
| Lake Study (6) | 2 | 1 | 1 | 3 | 1 | 2 | **2** |
| M98-863* (50) | 2 | 1 | 1 | 1 | 1 | 1 | **1** |
| METABOLIK (51) | 2 | 1 | 3 | 3 | 1 | 1 | **2** |
| Montaner 2006* (53) | 2 | 1 | 1 | 3 | 1 | 2 | **2** |
| NORTHIV (5) | 1 | 1 | 1 | 3 | 1 | 1 | **2** |
| Sierra-Madero 2010^†^ (54) | 2 | 1 | 1 | 3 | 1 | 2 | 2 |
| SINGLE (55) | 2 | 1 | 1 | 1 | 1 | 1 | **1** |
| SPRING-2 (56) | 2 | 1 | 1 | 1 | 1 | 1 | **1** |
| Squires 2004* (65); Jemsek 2006 (57) | 1 | 1 | 1 | 1 | 1 | 1 | **1** |
| STaR  (GS-US-264-0110) (58) | 1 | 1 | 1 | 3 | 1 | 1 | **2** |
| STARMRK (61) | 1 | 1 | 1 | 1 | 1 | 1 | **1** |
| THRIVE (64) | 1 | 1 | 1 | 1 | 1 | 1 | **1** |

*Connector studies; ^†^studies included in the subgroup analyses of viral suppression only; ^‡^considered representative of the ≤200 CD4^+^ cell/µL subgroup.

EPHPP, Effective Public Health Practice Project Quality Assessment; NMA, network meta-analysis.

**Supplementary Table 4: Summary of GRADE assessments for DTG comparisons with other core agents**

| **DTG vs** | **VS** | **CD4^+^** |
| --- | --- | --- |
| BIC | High | High |
| EVG/c | High | High |
| RAL | High | High |
| ATV/r | High | High |
| DRV/r | High | High |
| LPV/r | Moderate | High |
| EFV | High | High |
| RPV | High | High |

ATV/r, ritonavir-boosted atazanavir; BIC, bictegravir; DRV/r, ritonavir-boosted darunavir; DTG, dolutegravir; EFV, efavirenz; EVG/c, cobicistat-boosted elvitegravir; GRADE: Grading of Recommendations Assessment, Development and Evaluation; LPV/r, lopinavir-boosted ritonavir; RAL, raltegravir; RPV, rilpivirine.

**Supplementary Figure 1: Comparison of subgroup analysis of median change in VS at Week 48 by VL at baseline (≤ or >500,000 RNA copies/mL) with DTG versus core agents of interest (NRTI-unadjusted, FE model, log scale)**


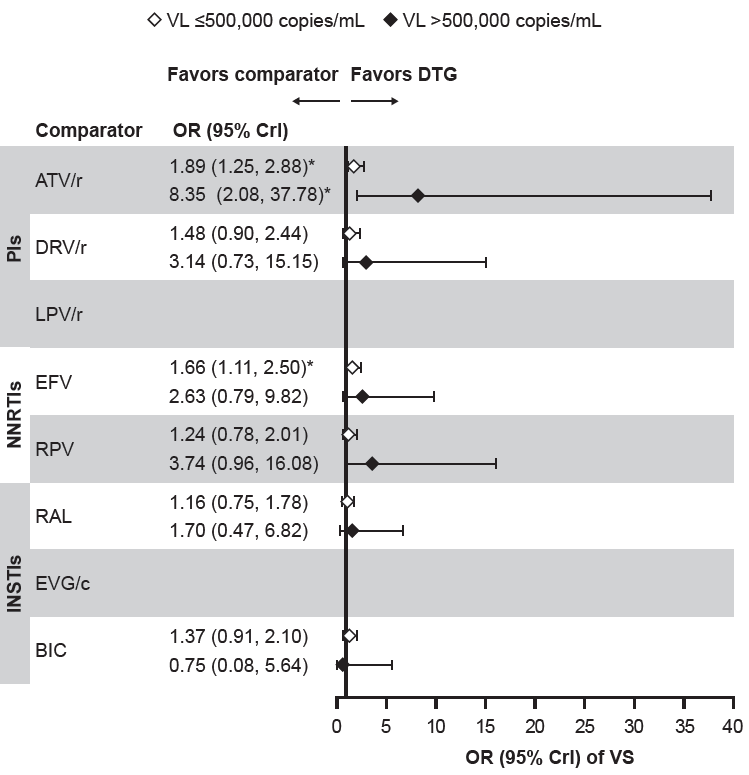


*Indicates treatment comparisons are significantly different. Two studies (both using BIC), classified patients according to baseline VL ≤400,000 and >400,000 copies/mL; for the purposes of this analysis these patients were included in the groups with baseline VL ≤500,000 and >500,000 copies/mL, respectively.

ATV/r, ritonavir-boosted atazanavir; BIC, bictegravir; Crl, credible interval; DTG, dolutegravir; DRV/r, ritonavir-boosted darunavir; EFV, efavirenz; EVG/c, cobicistat-boosted elvitegravir; FE, fixed effect; INSTIs, Integrase strand inhibitors; LPV/r, lopinavir-boosted ritonavir; NNRTIs, non-nucleoside reverse transcriptase inhibitors; NRTI, nucleoside/nucleotide reverse transcriptase inhibitor; OR, odds ratio; PIs, protease inhibitors; RAL, raltegravir; RPV: rilpivirine; VL: viral load.

**References**

1. Puhan MA, Schunemann HJ, Murad MH, Li T, Brignardello-Petersen R, Singh JA, et al. A GRADE Working Group approach for rating the quality of treatment effect estimates from network meta-analysis. BMJ. 2014;349:g5630.

2. The Cochrane Collaboration. Cochrane Handbook for Systematic Reviews of Interventions Version 5.1.0 [updated March 2011].

3. Dias S, Welton NJ, Sutton AJ, Caldwell DM, Lu G, Ades AE. Evidence synthesis for decision making 4: inconsistency in networks of evidence based on randomized controlled trials. Med Decis Making. 2013;33(5):641-56.

4. Puls RL, Srasuebkul P, Petoumenos K, Boesecke C, Duncombe C, Belloso WH, et al. Efavirenz versus boosted atazanavir or zidovudine and abacavir in antiretroviral treatment-naive, HIV-infected subjects: week 48 data from the Altair study. Clin Infect Dis. 2010;51(7):855-64.

5. Andersson LM, Vesterbacka J, Blaxhult A, Flamholc L, Nilsson S, Ormaasen V, et al. Lopinavir/ritonavir, atazanavir/ritonavir, and efavirenz in antiretroviral-naive HIV-1-infected individuals over 144 weeks: an open-label randomized controlled trial. Scand J Infect Dis. 2013;45(7):543-51.

6. Echeverria P, Negredo E, Carosi G, Galvez J, Gomez JL, Ocampo A, et al. Similar antiviral efficacy and tolerability between efavirenz and lopinavir/ritonavir, administered with abacavir/lamivudine (Kivexa), in antiretroviral-naive patients: a 48-week, multicentre, randomized study (Lake Study). Antiviral Res. 2010;85(2):403-8.

7. Patel DA, Snedecor SJ, Tang WY, Sudharshan L, Lim JW, Cuffe R, et al. 48-week efficacy and safety of dolutegravir relative to commonly used third agents in treatment-naive HIV-1-infected patients: a systematic review and network meta-analysis. PLoS One. 2014;9(9):e105653.

8. Molina JM, Andrade-Villanueva J, Echevarria J, Chetchotisakd P, Corral J, David N, et al. Once-daily atazanavir/ritonavir versus twice-daily lopinavir/ritonavir, each in combination with tenofovir and emtricitabine, for management of antiretroviral-naive HIV-1-infected patients: 48 week efficacy and safety results of the CASTLE study. Lancet. 2008;372(9639):646-55.

9. Gallant J, Lazzarin A, Mills A, Orkin C, Podzamczer D, Tebas P, et al. Bictegravir, emtricitabine, and tenofovir alafenamide versus dolutegravir, abacavir, and lamivudine for initial treatment of HIV-1 infection (GS-US-380-1489): a double-blind, multicentre, phase 3, randomised controlled non-inferiority trial. Lancet. 2017;390(10107):2063-72.

10. Sax PE, Pozniak A, Montes ML, Koenig E, DeJesus E, Stellbrink HJ, et al. Coformulated bictegravir, emtricitabine, and tenofovir alafenamide versus dolutegravir with emtricitabine and tenofovir alafenamide, for initial treatment of HIV-1 infection (GS-US-380-1490): a randomised, double-blind, multicentre, phase 3, non-inferiority trial. Lancet. 2017;390(10107):2073-82.

11. Malan DR, Krantz E, David N, Rong Y, Mathew M, Iloeje UH, et al. 96-week efficacy and safety of atazanavir, with and without ritonavir, in a HAART regimen in treatment-naive patients. J Int Assoc Physicians AIDS Care (Chic). 2010;9(1):34-42.

12. Malan DR, Krantz E, David N, Wirtz V, Hammond J, McGrath D, et al. Efficacy and safety of atazanavir, with or without ritonavir, as part of once-daily highly active antiretroviral therapy regimens in antiretroviral-naive patients. J Acquir Immune Defic Syndr. 2008;47(2):161-7.

13. van Leth F, Phanuphak P, Ruxrungtham K, Baraldi E, Miller S, Gazzard B, et al. Comparison of first-line antiretroviral therapy with regimens including nevirapine, efavirenz, or both drugs, plus stavudine and lamivudine: a randomised open-label trial, the 2NN Study. Lancet. 2004;363(9417):1253-63.

14. van Leth F, Phanuphak P, Stroes E, Gazzard B, Cahn P, Raffi F, et al. Nevirapine and efavirenz elicit different changes in lipid profiles in antiretroviral-therapy-naive patients infected with HIV-1. PLoS Med. 2004;1(1):e19.

15. ClinicalTrials.gov. Tenofovir Disoproxil Fumarate/Emtricitabine/Efavirenz Versus Combivir/Efavirenz in Antiretroviral-Naive HIV-1 Infected Subjects (NCT00112047) [updated 13 October 2010. Available from: <https://clinicaltrials.gov/ct2/show/results/NCT00112047>.

16. Gallant JE, DeJesus E, Arribas JR, Pozniak AL, Gazzard B, Campo RE, et al. Tenofovir DF, emtricitabine, and efavirenz vs. zidovudine, lamivudine, and efavirenz for HIV. N Engl J Med. 2006;354(3):251-60.

17. Pozniak AL, Gallant JE, DeJesus E, Arribas JR, Gazzard B, Campo RE, et al. Tenofovir disoproxil fumarate, emtricitabine, and efavirenz versus fixed-dose zidovudine/lamivudine and efavirenz in antiretroviral-naive patients: virologic, immunologic, and morphologic changes--a 96-week analysis. J Acquir Immune Defic Syndr. 2006;43(5):535-40.

18. Haubrich RH, Riddler SA, DiRienzo AG, Komarow L, Powderly WG, Klingman K, et al. Metabolic outcomes in a randomized trial of nucleoside, nonnucleoside and protease inhibitor-sparing regimens for initial HIV treatment. AIDS. 2009;23(9):1109-18.

19. Riddler SA, Haubrich R, DiRienzo AG, Peeples L, Powderly WG, Klingman KL, et al. Class-sparing regimens for initial treatment of HIV-1 infection. N Engl J Med. 2008;358(20):2095-106.

20. Daar ES, Tierney C, Fischl MA, Sax PE, Mollan K, Budhathoki C, et al. Atazanavir plus ritonavir or efavirenz as part of a 3-drug regimen for initial treatment of HIV-1. Ann Intern Med. 2011;154(7):445-56.

21. Smith KY, Weinberg WG, DeJesus E, Fischl MA, Liao Q, Ross LL, et al. Fosamprenavir or atazanavir once daily boosted with ritonavir 100 mg, plus tenofovir/emtricitabine, for the initial treatment of HIV infection: 48-week results of ALERT. AIDS Res Ther. 2008;5:5.

22. Orrell C, Hagins DP, Belonosova E, Porteiro N, Walmsley S, Falco V, et al. Fixed-dose combination dolutegravir, abacavir, and lamivudine versus ritonavir-boosted atazanavir plus tenofovir disoproxil fumarate and emtricitabine in previously untreated women with HIV-1 infection (ARIA): week 48 results from a randomised, open-label, non-inferiority, phase 3b study. Lancet HIV. 2017;4(12):e536-e46.

23. Lathouwers E, De Meyer S, Dierynck I, Van de Casteele T, Lavreys L, de Bethune MP, et al. Virological characterization of patients failing darunavir/ritonavir or lopinavir/ritonavir treatment in the ARTEMIS study: 96-week analysis. Antivir Ther. 2011;16(1):99-108.

24. Mills AM, Nelson M, Jayaweera D, Ruxrungtham K, Cassetti I, Girard PM, et al. Once-daily darunavir/ritonavir vs. lopinavir/ritonavir in treatment-naive, HIV-1-infected patients: 96-week analysis. AIDS. 2009;23(13):1679-88.

25. Ortiz R, Dejesus E, Khanlou H, Voronin E, van Lunzen J, Andrade-Villanueva J, et al. Efficacy and safety of once-daily darunavir/ritonavir versus lopinavir/ritonavir in treatment-naive HIV-1-infected patients at week 48. AIDS. 2008;22(12):1389-97.

26. Moyle GJ, Stellbrink HJ, Compston J, Orkin C, Arribas JR, Domingo P, et al. 96-Week results of abacavir/lamivudine versus tenofovir/emtricitabine, plus efavirenz, in antiretroviral-naive, HIV-1-infected adults: ASSERT study. Antivir Ther. 2013;18(7):905-13.

27. Post FA, Moyle GJ, Stellbrink HJ, Domingo P, Podzamczer D, Fisher M, et al. Randomized comparison of renal effects, efficacy, and safety with once-daily abacavir/lamivudine versus tenofovir/emtricitabine, administered with efavirenz, in antiretroviral-naive, HIV-1-infected adults: 48-week results from the ASSERT study. J Acquir Immune Defic Syndr. 2010;55(1):49-57.

28. Martinez E, Gonzalez-Cordon A, Ferrer E, Domingo P, Negredo E, Gutierrez F, et al. Early lipid changes with atazanavir/ritonavir or darunavir/ritonavir. HIV Med. 2014;15(6):330-8.

29. Vrouenraets SM, Wit FW, Fernandez Garcia E, Moyle GJ, Jackson AG, Allavena C, et al. Randomized comparison of metabolic and renal effects of saquinavir/r or atazanavir/r plus tenofovir/emtricitabine in treatment-naive HIV-1-infected patients. HIV Med. 2011;12(10):620-31.

30. ClinicalTrials.gov. TMC278-TiDP6-C209: A Clinical Trial in Treatment Naive HIV-1 Patients Comparing TMC278 to Efavirenz in Combination With Tenofovir + Emtricitabine (NCT00540449) [updated 29 March 2016. Available from: <https://clinicaltrials.gov/ct2/show/results/NCT00540449>.

31. Molina JM, Andrade-Villanueva J, Echevarria J, Chetchotisakd P, Corral J, David N, et al. Once-daily atazanavir/ritonavir compared with twice-daily lopinavir/ritonavir, each in combination with tenofovir and emtricitabine, for management of antiretroviral-naive HIV-1-infected patients: 96-week efficacy and safety results of the CASTLE study. J Acquir Immune Defic Syndr. 2010;53(3):323-32.

32. Uy J, Yang R, Wirtz V, Sheppard L, Farajallah A, McGrath D. Treatment of advanced HIV disease in antiretroviral-naive HIV-1-infected patients receiving once-daily atazanavir/ritonavir or twice-daily lopinavir/ritonavir, each in combination with tenofovir disoproxil fumarate and emtricitabine. AIDS Care. 2011;23(11):1500-4.

33. Bartlett JA, Johnson J, Herrera G, Sosa N, Rodriguez A, Liao Q, et al. Long-term results of initial therapy with abacavir and Lamivudine combined with Efavirenz, Amprenavir/Ritonavir, or Stavudine. J Acquir Immune Defic Syndr. 2006;43(3):284-92.

34. Clumeck N, Mwamba C, Kabeya K, Matanda S, Vaira D, Necsoi C, et al. First-line antiretroviral therapy with nevirapine versus lopinavir-ritonavir based regimens in a resource-limited setting. AIDS. 2014;28(8):1143-53.

35. DeJesus E, Herrera G, Teofilo E, Gerstoft J, Buendia CB, Brand JD, et al. Abacavir versus zidovudine combined with lamivudine and efavirenz, for the treatment of antiretroviral-naive HIV-infected adults. Clin Infect Dis. 2004;39(7):1038-46.

36. Molina JM, Cahn P, Grinsztejn B, Lazzarin A, Mills A, Saag M, et al. Rilpivirine versus efavirenz with tenofovir and emtricitabine in treatment-naive adults infected with HIV-1 (ECHO): a phase 3 randomised double-blind active-controlled trial. Lancet. 2011;378(9787):238-46.

37. Nelson M, Amaya G, Clumeck N, Arns da Cunha C, Jayaweera D, Junod P, et al. Efficacy and safety of rilpivirine in treatment-naive, HIV-1-infected patients with hepatitis B virus/hepatitis C virus coinfection enrolled in the Phase III randomized, double-blind ECHO and THRIVE trials. J Antimicrob Chemother. 2012;67(8):2020-8.

38. Clotet B, Feinberg J, van Lunzen J, Khuong-Josses MA, Antinori A, Dumitru I, et al. Once-daily dolutegravir versus darunavir plus ritonavir in antiretroviral-naive adults with HIV-1 infection (FLAMINGO): 48 week results from the randomised open-label phase 3b study. Lancet. 2014;383(9936):2222-31.

39. ClinicalTrials.gov. Abacavir/Lamivudine Versus Emtricitabine/Tenofovir Both In Combination With Lopinavir/Ritonavir For The Treatment Of HIV (HEAT) (NCT00244712) [updated 8 June 2010. Available from: <https://clinicaltrials.gov/ct2/show/results/NCT00244712>.

40. Sax PE, DeJesus E, Mills A, Zolopa A, Cohen C, Wohl D, et al. Co-formulated elvitegravir, cobicistat, emtricitabine, and tenofovir versus co-formulated efavirenz, emtricitabine, and tenofovir for initial treatment of HIV-1 infection: a randomised, double-blind, phase 3 trial, analysis of results after 48 weeks. Lancet. 2012;379(9835):2439-48.

41. Zolopa A, Sax PE, DeJesus E, Mills A, Cohen C, Wohl D, et al. A randomized double-blind comparison of coformulated elvitegravir/cobicistat/emtricitabine/tenofovir disoproxil fumarate versus efavirenz/emtricitabine/tenofovir disoproxil fumarate for initial treatment of HIV-1 infection: analysis of week 96 results. J Acquir Immune Defic Syndr. 2013;63(1):96-100.

42. DeJesus E, Rockstroh JK, Henry K, Molina JM, Gathe J, Ramanathan S, et al. Co-formulated elvitegravir, cobicistat, emtricitabine, and tenofovir disoproxil fumarate versus ritonavir-boosted atazanavir plus co-formulated emtricitabine and tenofovir disoproxil fumarate for initial treatment of HIV-1 infection: a randomised, double-blind, phase 3, non-inferiority trial. Lancet. 2012;379(9835):2429-38.

43. Rockstroh JK, DeJesus E, Lennox JL, Yazdanpanah Y, Saag MS, Wan H, et al. Durable efficacy and safety of raltegravir versus efavirenz when combined with tenofovir/emtricitabine in treatment-naive HIV-1-infected patients: final 5-year results from STARTMRK. J Acquir Immune Defic Syndr. 2013;63(1):77-85.

44. Canadian Drug Expert Committee. CADTH Canadian Drug Expert Committee Recommendation – BICTEGRAVIR/EMTRICITABINE/TENOFOVIR ALAFENAMIDE (BIKTARVY — GILEAD SCIENCES CANADA, INC.) 2018 [updated October 2018. Available from: <https://www.cadth.ca/sites/default/files/cdr/complete/SR0567%20Biktarvy%20-%20CDEC%20Final%20Recommendation%20October%2029%2C%202018.pdf>.

45. Smith KY, Patel P, Fine D, Bellos N, Sloan L, Lackey P, et al. Randomized, double-blind, placebo-matched, multicenter trial of abacavir/lamivudine or tenofovir/emtricitabine with lopinavir/ritonavir for initial HIV treatment. AIDS. 2009;23(12):1547-56.

46. Slama L, Landman R, Assoumou L, Benalycherif A, Samri A, Joly V, et al. Efficacy and safety of once-daily ritonavir-boosted atazanavir or darunavir in combination with a dual nucleos(t)ide analogue backbone in HIV-1-infected combined ART (cART)-naive patients with severe immunosuppression: a 48 week, non-comparative, randomized, multicentre trial (IMEA 040 DATA trial). J Antimicrob Chemother. 2016;71(8):2252-61.

47. Initio Trial International Co-ordinating Committee, Yeni P, Cooper DA, Aboulker JP, Babiker AG, Carey D, et al. Virological and immunological outcomes at 3 years after starting antiretroviral therapy with regimens containing non-nucleoside reverse transcriptase inhibitor, protease inhibitor, or both in INITIO: open-label randomised trial. Lancet. 2006;368(9532):287-98.

48. Eron J, Jr., Yeni P, Gathe J, Jr., Estrada V, DeJesus E, Staszewski S, et al. The KLEAN study of fosamprenavir-ritonavir versus lopinavir-ritonavir, each in combination with abacavir-lamivudine, for initial treatment of HIV infection over 48 weeks: a randomised non-inferiority trial. Lancet. 2006;368(9534):476-82.

49. Pulido F, Estrada V, Baril JG, Logue K, Schewe K, Plettenberg A, et al. Long-term efficacy and safety of fosamprenavir plus ritonavir versus lopinavir/ritonavir in combination with abacavir/lamivudine over 144 weeks. HIV Clin Trials. 2009;10(2):76-87.

50. Walmsley S, Bernstein B, King M, Arribas J, Beall G, Ruane P, et al. Lopinavir-ritonavir versus nelfinavir for the initial treatment of HIV infection. N Engl J Med. 2002;346(26):2039-46.

51. Aberg JA, Tebas P, Overton ET, Gupta SK, Sax PE, Landay A, et al. Metabolic effects of darunavir/ritonavir versus atazanavir/ritonavir in treatment-naive, HIV type 1-infected subjects over 48 weeks. AIDS Res Hum Retroviruses. 2012;28(10):1184-95.

52. ClinicalTrials.gov. TMC278-TiDP6-C215: A Clinical Trial in Treatment Naive HIV-subjects Patients Comparing TMC278 to Efavirenz in Combination With 2 Nucleoside/Nucleotide Reverse Transcriptase Inhibitors (NCT00543725) [updated 1 April 2016. Available from: <https://clinicaltrials.gov/ct2/show/results/NCT00543725>.

53. Montaner JS, Schutz M, Schwartz R, Jayaweera DT, Burnside AF, Walmsley S, et al. Efficacy, safety and pharmacokinetics of once-daily saquinavir soft-gelatin capsule/ritonavir in antiretroviral-naive, HIV-infected patients. MedGenMed. 2006;8(2):36.

54. Sierra-Madero J, Villasis-Keever A, Mendez P, Mosqueda-Gomez JL, Torres-Escobar I, Gutierrez-Escolano F, et al. Prospective, randomized, open label trial of Efavirenz vs Lopinavir/Ritonavir in HIV+ treatment-naive subjects with CD4+<200 cell/mm3 in Mexico. J Acquir Immune Defic Syndr. 2010;53(5):582-8.

55. Walmsley SL, Antela A, Clumeck N, Duiculescu D, Eberhard A, Gutierrez F, et al. Dolutegravir plus abacavir-lamivudine for the treatment of HIV-1 infection. N Engl J Med. 2013;369(19):1807-18.

56. Raffi F, Rachlis A, Stellbrink HJ, Hardy WD, Torti C, Orkin C, et al. Once-daily dolutegravir versus raltegravir in antiretroviral-naive adults with HIV-1 infection: 48 week results from the randomised, double-blind, non-inferiority SPRING-2 study. Lancet. 2013;381(9868):735-43.

57. Jemsek JG, Arathoon E, Arlotti M, Perez C, Sosa N, Pokrovskiy V, et al. Body fat and other metabolic effects of atazanavir and efavirenz, each administered in combination with zidovudine plus lamivudine, in antiretroviral-naive HIV-infected patients. Clin Infect Dis. 2006;42(2):273-80.

58. Cohen C, Wohl D, Arribas JR, Henry K, Van Lunzen J, Bloch M, et al. Week 48 results from a randomized clinical trial of rilpivirine/emtricitabine/tenofovir disoproxil fumarate vs. efavirenz/emtricitabine/tenofovir disoproxil fumarate in treatment-naive HIV-1-infected adults. AIDS. 2014;28(7):989-97.

59. van Lunzen J, Antinori A, Cohen CJ, Arribas JR, Wohl DA, Rieger A, et al. Rilpivirine vs. efavirenz-based single-tablet regimens in treatment-naive adults: week 96 efficacy and safety from a randomized phase 3b study. AIDS. 2016;30(2):251-9.

60. Lennox JL, Dejesus E, Berger DS, Lazzarin A, Pollard RB, Ramalho Madruga JV, et al. Raltegravir versus Efavirenz regimens in treatment-naive HIV-1-infected patients: 96-week efficacy, durability, subgroup, safety, and metabolic analyses. J Acquir Immune Defic Syndr. 2010;55(1):39-48.

61. Lennox JL, DeJesus E, Lazzarin A, Pollard RB, Madruga JV, Berger DS, et al. Safety and efficacy of raltegravir-based versus efavirenz-based combination therapy in treatment-naive patients with HIV-1 infection: a multicentre, double-blind randomised controlled trial. Lancet. 2009;374(9692):796-806.

62. Rockstroh J, Teppler H, Zhao J, Sklar P, Harvey C, Strohmaier K, et al. Safety and efficacy of raltegravir in patients with HIV-1 and hepatitis B and/or C virus coinfection. HIV Med. 2012;13(2):127-31.

63. ClinicalTrials.gov. A Study to Evaluate the Safety and Antiretroviral Activity of MK-0518 Versus Efavirenz in Treatment Naive HIV-Infected Patients, Each in Combination With TRUVADA (0518-021 EXT) (NCT00369941) [updated 21 March. Available from: <https://clinicaltrials.gov/ct2/show/results/NCT00369941>.

64. Cohen CJ, Andrade-Villanueva J, Clotet B, Fourie J, Johnson MA, Ruxrungtham K, et al. Rilpivirine versus efavirenz with two background nucleoside or nucleotide reverse transcriptase inhibitors in treatment-naive adults infected with HIV-1 (THRIVE): a phase 3, randomised, non-inferiority trial. Lancet. 2011;378(9787):229-37.

65. Squires K, Lazzarin A, Gatell JM, Powderly WG, Pokrovskiy V, Delfraissy JF, et al. Comparison of once-daily atazanavir with efavirenz, each in combination with fixed-dose zidovudine and lamivudine, as initial therapy for patients infected with HIV. J Acquir Immune Defic Syndr. 2004;36(5):1011-9.
